# When larger brains do not have more neurons: increased numbers of cells are compensated by decreased average cell size across mouse individuals

**DOI:** 10.3389/fnana.2015.00064

**Published:** 2015-06-01

**Authors:** Suzana Herculano-Houzel, Débora J. Messeder, Karina Fonseca-Azevedo, Nilma A. Pantoja

**Affiliations:** Instituto de Ciências Biomédicas, Universidade Federal do Rio de Janeiro and Instituto Nacional de Neurociência Translacional, MCT/INCTRio de Janeiro, Brazil

**Keywords:** intraspecific variation, brain size, number of neurons, neurons, number of glia, neuronal density

## Abstract

There is a strong trend toward increased brain size in mammalian evolution, with larger brains composed of more and larger neurons than smaller brains across species within each mammalian order. Does the evolution of increased numbers of brain neurons, and thus larger brain size, occur simply through the selection of individuals with more and larger neurons, and thus larger brains, within a population? That is, do individuals with larger brains also have more, and larger, neurons than individuals with smaller brains, such that allometric relationships across species are simply an extension of intraspecific scaling? Here we show that this is not the case across adult male mice of a similar age. Rather, increased numbers of neurons across individuals are accompanied by increased numbers of other cells and smaller average cell size of both types, in a trade-off that explains how increased brain mass does not necessarily ensue. Fundamental regulatory mechanisms thus must exist that tie numbers of neurons to numbers of other cells and to average cell size within individual brains. Finally, our results indicate that changes in brain size in evolution are not an extension of individual variation in numbers of neurons, but rather occur through step changes that must simultaneously increase numbers of neurons and cause cell size to increase, rather than decrease.

## Introduction

Evolution—whose occurrence is evident in the morphological and genetic variations that characterize different species—is considered to feed on the raw material of intraspecific variation, shaped over generations by selective forces. It is therefore remarkable that in the evolution of mammalian brains, which have tended to increase in mass over the last 65 million years (Jerison, 1973), the allometric relationships that apply across species do not also apply across individuals of a single species. As reviewed by Armstrong (1990), while mammalian species with larger bodies tend to have larger brains, larger individuals of a same species do not necessarily have larger brains—or do so with a much smaller allometric exponent (Kruska, 2007).

We have shown that larger mammalian brains, varying in mass over 10,000 times across species, have more neurons than smaller brains within each mammalian order, and across non-primate species, the more the neurons, the larger the average size of neuronal cells (including all dendritic and axonal arbors; Herculano-Houzel et al., 2014). The simplest mechanism to explain the evolutionary origin of brains with increased numbers of neurons associated with increased average neuronal cell size (and hence decreased average neuronal density; Mota and Herculano-Houzel, 2014) would be if they were the result of positive selection of those individuals within populations that carried the most neurons associated with decreased neuronal densities, and thus the largest brains. This mechanism presupposes that, across individuals as across species, increased numbers of neurons are associated with increased average neuronal cell size (and hence decreased neuronal density) in the same manner, such that interspecific allometric relationships between brain size and number of neurons are simply an extension of those relationships across individuals of a single species.

However, given that allometric relationships across species are not necessarily reproduced at the intraspecific level (Armstrong, 1990), there is no *a priori* reason to expect animals with larger brains within a species to also have more neurons. If differences across species turn out not to be an exaggeration of differences across individuals of a same species, then how does evolution create great diversity and strong allometric relationships across species from weak or inexistent allometric relationships across individuals?

The issue of intraspecific variation in the relationship between brain size and number of neurons is also of great potential importance given that, on the one hand, variation in absolute cortical (brain) size is the best predictor of a species' cognitive abilities within an order (Deaner et al., 2007), and on the other hand, intraspecific variation in brain size shows a fair degree of correlation with cognitive abilities (Luders et al., 2009). It is thus a pressing question to answer: do individuals with larger brains also have more neurons—and, ultimately, does this correlate with individual variations in cognitive abilities?

Here we use the isotropic fractionator (Herculano-Houzel and Lent, 2005), which gives comparable results to stereology but in less time (Bahney and von Bartheld, 2014; Miller et al., 2014), to determine whether intraspecific variation in the size of CNS structures (brain and spinal cord) across individuals of the non-isogenic Swiss strain of mice (*Mus musculus*) is correlated with variation in the number of neurons and other cells that compose these structures as well as in the average cell size of neurons and other cells, inferred from neuronal and other cell densities in the different structures.

## Material and methods

We analyzed the brain and spinal cord of 19 male mice of the Swiss variety, a non-isogenic strain. All animals were born in the same week, and were 2 months old at the time of death. All procedures were carried out in conformity with NIH, and Society for Neuroscience guidelines as well as with those of the Committee for the Ethical Use of Animals of the University. Animals were killed by inhalation of an overdose of ether, and perfused through the heart with a 0.9% saline solution followed by 4% phosphate-buffered paraformaldehyde. The brain was removed, sectioned sagittally, and dissected into cerebral cortex (including the hippocampus), cerebellum, olfactory bulb, and remaining areas (the ensemble of brainstem, diencephalon and basal ganglia). No tissue was left unaccounted for; the entirety of each brain is contained in the dissected cerebral cortex, cerebellum, olfactory bulb, and remaining areas. The left and right halves of each brain structure were processed separately. The spinal cord was also collected from all animals, from the foramen magnum to the proximal limit of the *cauda equina*. All structures were weighed immediately after dissection upon removal of the dura mater and the superficial vasculature.

After 2–3 weeks of post-fixation, all brain structures and spinal cords were processed using the isotropic fractionator to determine the numbers of neuronal and other cells that composed them (Herculano-Houzel and Lent, 2005; Herculano-Houzel, 2012). This method consists of dissociating brain tissue into a suspension of free cell nuclei, which can be counted by sampling after the suspension is made homogeneous (isotropic) by agitation, followed by the determination of the proportion of nuclei that belonged to neurons according to the expression of the neuronal marker NeuN detected by immunocytochemistry. Each structure was dissociated mechanically in a 1% Triton X-100 solution in 40 mM sodium citrate; the suspension containing all free cell nuclei was stained with DAPI diluted 20–50 × from a stock solution of 20 mg/mL, and rounded up to a known volume; and the density of cell nuclei in the suspension was determined from counting four aliquots of the suspension under a fluorescence microscope. This yielded estimates of total numbers of cells in each structure with a within-sample CV of typically below 0.10. One sample of the nuclear suspension for each structure was then reacted with anti-NeuN antibody (Millipore mab377), an appropriate secondary antibody, and scored under the microscope for the percentage of NeuN-positive nuclei within a total of at least 500 nuclei. The isotropic fractionator method has been described in detail elsewhere (Herculano-Houzel, 2012), and shown independently by two other groups to yield results similar to those obtained with stereology (Bahney and von Bartheld, 2014; Miller et al., 2014; Herculano-Houzel et al., 2015).

For consistency and ease of comparison with our previous publications on rodent brains (Herculano-Houzel et al., 2006, 2011), “total brain” mass and numbers of cells do not include the olfactory bulb (that is, they are the sum of cerebral cortex, cerebellum, and remaining areas).

Numbers of neurons were determined directly with the isotropic fractionator for each tissue in each dissected structure; numbers of other cells were determined by subtraction of the number of neurons from the total number of cells in each structure. Due to the lack of suitable universal nuclear markers to identify oligodendrocytes, astrocytes, microglia and endothelial cells, all of these cell types are lumped together as “other cells”, the majority of which is presumed to be glial cell types, given that the endothelium occupies only 1% of the cortical volume in mice (Tsai et al., 2009). Densities of either cell type were calculated as the simple ratio between the number of neuronal (or other cells) in the tissue and the mass of the dissected tissue (in mg). As shown before, densities are inversely correlated with average cell size in the tissue, and can therefore be used as proxies for the inverse of average cell size: the larger the density of cells in the tissue, the smaller the average cell size (Mota and Herculano-Houzel, 2014). Notice that this average cell size makes no distinction across cell compartments, and refers necessarily to the ensemble of cell body and dendritic and axonal arbors in the dissected tissue.

All statistical analyses were performed in JMP 9.0 (SAS, USA). Comparisons between left and right brain structures were made using Wilcoxon's test and matched pair analysis for each structure. Pairwise correlations were between variables calculated using Spearman rank correlation, a non-parametric analysis that makes no assumptions about normality in the dataset. Power laws were fit to natural log-transformed data. A principal component analysis was performed using as variables structure mass, numbers of cells, densities and ratio between numbers of other cells and neurons (O/N ratio) across all structures.

## Results

**Figure 1:**
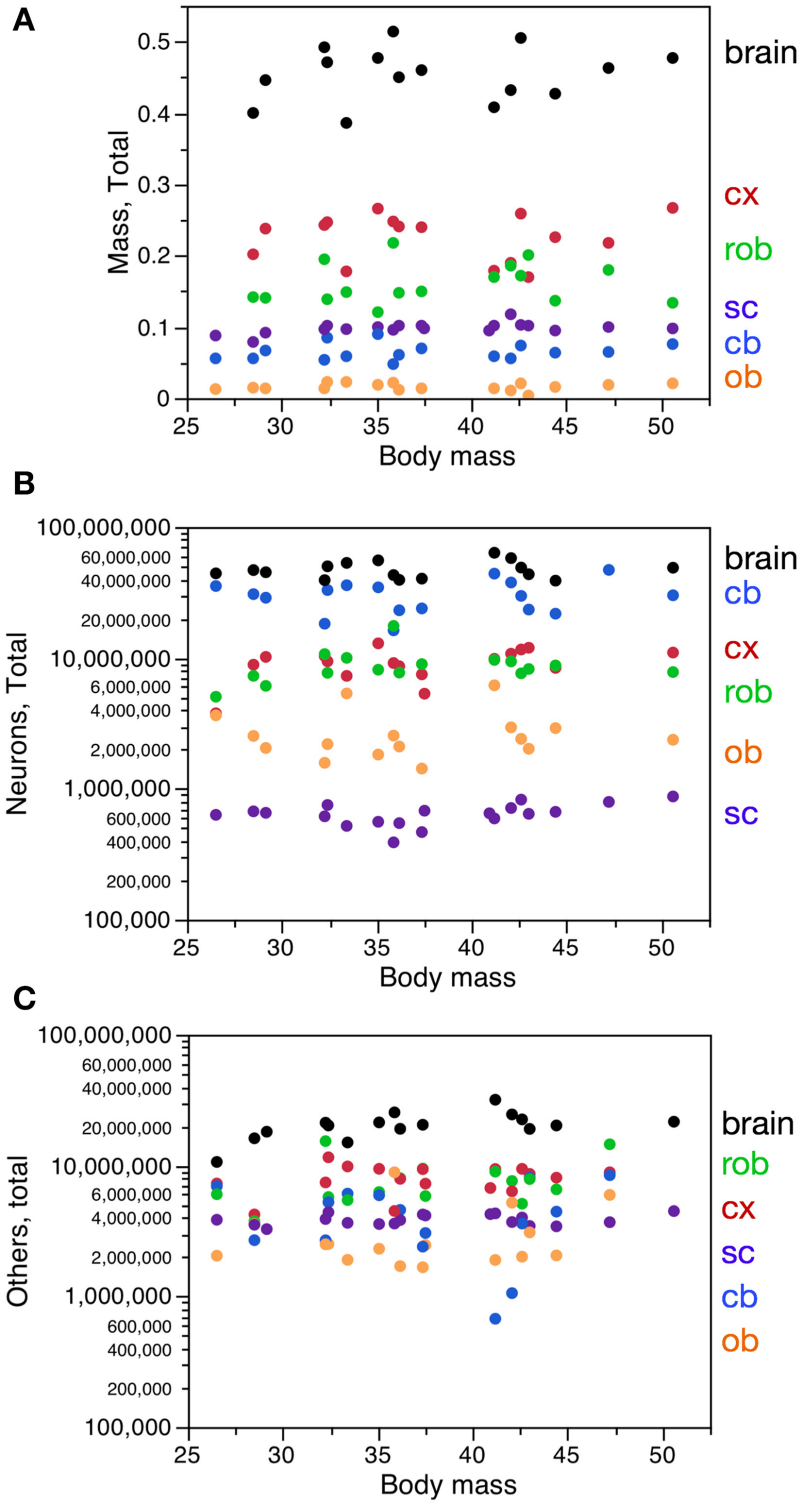
**Variation in CNS structure mass, number of neurons and number of other cells with body mass across mouse individuals**. Each data point represents one CNS structure in one individual. Cx, cerebral cortex (gray and white matter combined); cb, cerebellum; ob, olfactory bulb; rob, rest of brain; sc, spinal cord; brain, whole brain excluding the olfactory bulb. All values refer to structures in both sides of the brain together. **(A)** CNS structure mass. **(B)** Total number of neurons in each CNS structure. **(C)** Total number of other cells in each CNS structure.

The 19 male mouse individuals of similar age (2 months, all born within the same week) varied by 1.91-fold in body mass, 1.33-fold in brain mass, 1.63-fold in number of brain neurons, and 2.98-fold in number of other cells in the brain (Figure 1). Across these individuals, brain structure mass, brain mass and spinal cord mass had a range of variation between 1.33-fold and 3.50-fold, depending on the structure, while numbers of neurons in these structures had a slightly larger range of variation (1.63- to 4.53-fold), but larger coefficients of variation (CV) than structure mass (Table 1). Numbers of other cells in each brain structure, brain or spinal cord varied across individuals with a range of variation between 1.37 and 12.51-fold, with CVs similar to those found for numbers of neurons (Table 1).

**Table 1:** **Average structure mass and number of cells in each brain and spinal cord**.

**Structure mass**	**LH**	**RH**	**Total**
**CEREBRAL CORTEX**			
Average, g	0.113 ± 0.004	0.111 ± 0.004	0.226 ± 0.008
Minimum mass, g	0.085	0.080	0.170
Maximum mass, g	0.134	0.137	0.267
Variation	1.58 ×	1.71 ×	1.57 ×
**CEREBELLUM**			
Average, g	0.032 ± 0.002	0.033 ± 0.001	0.065 ± 0.003
Minimum mass, g	0.024	0.024	0.048
Maximum mass, g	0.050	0.044	0.090
Variation	2.08 ×	1.83 ×	1.88 ×
**OLFACTORY BULB**			
Average, g	0.008 ± 0.001	0.009 ± 0.001	0.017 ± 0.001
Minimum mass, g	0.004	0.004	0.011
Maximum mass, g	0.012	0.014	0.023
Variation	3.0 ×	3.5 ×	2.09 ×
**REMAINING AREAS**			
Average, g	0.080 ± 0.004	0.081 ± 0.003	0.161 ± 0.007
Minimum mass, g	0.054	0.062	0.121
Maximum mass, g	0.113	0.105	0.218
Variation	2.09 ×	1.69 ×	1.80 ×
**SPINAL CORD**			
Average, g			0.098 ± 0.002
Minimum mass, g			0.079
Maximum mass, g			0.118
Variation			1.49 ×
**WHOLE BRAIN**			
Average, g			0.454 ± 0.010
Minimum mass, g			0.386
Maximum mass, g			0.514
Variation			1.33 ×
**Numbers of neurons**	**LH**	**RH**	**Both**
**CEREBRAL CORTEX**			
Average, million	4.785 ± 0.226	5.027 ± 0.272	9.859 ± 0.418
Minimum, million	2.366	3.484	7.294
Maximum, million	6.011	7.225	12.900
Variation	2.54 ×	2.07 ×	1.77 ×
**CEREBELLUM**			
Average, million	16.901 ± 1.383	14.330 ± 1.048	30.664 ± 2.156
Minimum, million	8.432	7.815	16.200
Maximum, million	27.100	23.400	47.000
Variation	3.21 ×	2.99 ×	2.90 ×
**OLFACTORY BULB**			
Average, million	1.489 ± 0.199	1.360 ± 0.151	2.756 ± 0.326
Minimum, million	0.746	0.682	1.428
Maximum, million	3.376	2.804	6.180
Variation	4.53 ×	4.11 ×	4.33 ×
**REMAINING AREAS**			
Average, million	4.371 ± 0.363	4.700 ± 0.328	8.797 ± 0.684
Minimum, million	2.975	3.141	5.051
Maximum, million	8.746	8.832	17.600
Variation	2.94 ×	2.81 ×	3.48 ×
**SPINAL CORD**			
Average, million			0.645 ± 0.027
Minimum, million			0.390
Maximum, million			0.874
Variation			2.24 ×
**WHOLE BRAIN**			
Average, million			47.463 ± 1.809
Minimum, million			38.900
Maximum, million			63.600
Variation			1.63 ×
**Numbers of other cells**	**LH**	**RH**	**Both**
**CEREBRAL CORTEX**			
Average, million	4.402 ± 0.278	4.366 ± 0.196	8.252 ± 0.404
Minimum, million	2.443	3.219	4.224
Maximum, million	6.729	6.255	11.600
Variation	2.75 ×	1.94 ×	2.75 ×
**CEREBELLUM**			
Average, million	2.555 ± 0.371	2.011 ± 0.220	4.416 ± 0.541
Minimum, million	0.477	0.582	0.674
Maximum, million	6.024	3.494	8.431
Variation	12.64 ×	6.00 ×	12.51 ×
**OLFACTORY BULB**			
Average, millions	1.487 ± 0.199	1.336 ± 0.129	2.731 ± 0.308
Minimum, millions	0.834	0.830	1.670
Maximum, millions	3.514	2.446	5.960
Variation	4.21 ×	2.95 ×	3.57 ×
**REMAINING AREAS**			
Average, million	3.962 ± 0.456	4.019 ± 0.340	7.733 ± 0.795
Minimum, million	2.287	2.529	3.749
Maximum, million	8.204	7.168	15.400
Variation	3.59 ×	2.83 ×	4.11 ×
**SPINAL CORD**			
Average, million			3.855 ± 0.082
Minimum, million			3.264
Maximum, million			4.481
Variation			1.37 ×
**WHOLE BRAIN**			
Average, million			20.492 ± 1.177
Minimum, million			10.700
Maximum, million			31.900
Variation			2.98 ×

Values are averages ± SE. Variation refers to the ratio between maximum and minimum values for each parameter. Values for the whole brain do not include the olfactory bulb.

### No correlation with body mass

Variations in body mass and in brain mass (Figure 1) were not significantly correlated with each other across individuals (Spearman correlation, ρ = 0.1555, *p* = 0.5800). Variations in spinal cord mass across individuals were also not correlated with variations in body mass (ρ = 0.4788, *p* = 0.0381 across all individuals, but only if smallest spinal cord included; without this single data point, ρ = 0.3877, *p* = 0.1119). Variations in spinal cord mass were also not correlated with variations in brain mass across individuals (ρ = 0.1834, *p* = 0.5130). Thus, larger animals do not have larger brains or spinal cords, and larger brains are not associated with larger spinal cords.

Variations in body mass were not correlated with variations in the number of neurons in the brain or spinal cord (ρ = 0.0059, *p* = 0.9828 and ρ = 0.4193, *p* = 0.0739, respectively), nor with the numbers of other cells in these structures (ρ = 0.5471, *p* = 0.0283 and ρ = 0.1754, *p* = 0.4785, respectively, but correlation for the brain is only significant if smallest brain is included; without this single data point, ρ = 0.4500, *p* = 0.0924). Thus, larger animals do not have more neurons in the CNS, and at best have a weak tendency to have more other cells in the brain (Figures 1B,C).

### Differences across left and right brain structures

No overall significant differences were found across individuals between the left and right sides of brain structures in mass, number of neurons, number of other cells, or cell densities (Wilcoxon test, all values of *p* > 0.1). Additionally, values obtained for the left half of the brain correlated strongly with values obtained for the right half across structures (Spearman correlation, ρ > 0.8 and *p* < 0.0001 for mass, number of neurons, number of other cells, neuronal density, other cell density, and other cell/neuron ratio).

Matched pairs analysis revealed no overall significant differences in the mass of the structures across the two sides of the brain (all values of *p* > 0.05). However, there was a significantly larger number of neurons in one side of the cerebellum and olfactory bulb compared to the other. In both structures, the left side had significantly more neurons than the right side (mean difference, 3.100 ± 0.962 and 0.173 ± 0.092 million neurons, respectively, *p* = 0.0026 and 0.0397; cerebral cortex, *p* = 0.1259; rest of brain, *p* = 0.0848). The difference amounts to 18.3 and 11.6% of the neurons in the left side of the cerebellum and olfactory bulb, respectively. A significantly larger number of other cells in one side than the other was found only in the olfactory bulb, where again the left olfactory bulb had more other cells than the right bulb (mean difference, 188,163 ± 96,079 other cells, *p* = 0.0352), which amounts to 12.6% of the other cells in the left olfactory bulb (all other values of *p* > 0.05). Given that numbers of cells are supposedly determined independently in the two halves of the brain, the remaining analyses are performed jointly on data from the left and right halves for each structure (shown as open and filled symbols; *n* = 38 for 19 individuals, except where particular data are missing for an individual).

A principal component analysis of the variation in structure mass, numbers of cells, densities and ratio between numbers of other cells and neurons (O/N ratio) performed across all structures pooled together (that is, irrespective of structure identity) reveals two main components that account for 82% of the variation. The first component is loaded by number of neurons (loading factor, 0.948) followed by neuronal density (0.915), O/N ratio (−0.902) and total number of cells (0.849), and accounts for 49.2% of variation (eigenvalue, 3.444). The second component is loaded by structure mass (0.878), number of other cells (0.807), and other cell density (−0.667; eigenvalue, 2.280), and accounts cumulatively for 81.8% of the variation (eigenvalue, 2.2798). Importantly, the finding that structure mass only loads significantly in the second component suggests that it is a consequence, and not a determinant, of variation across individuals.

### Variations in structure mass are poorly correlated with cell numbers and density

**Figure 2:**
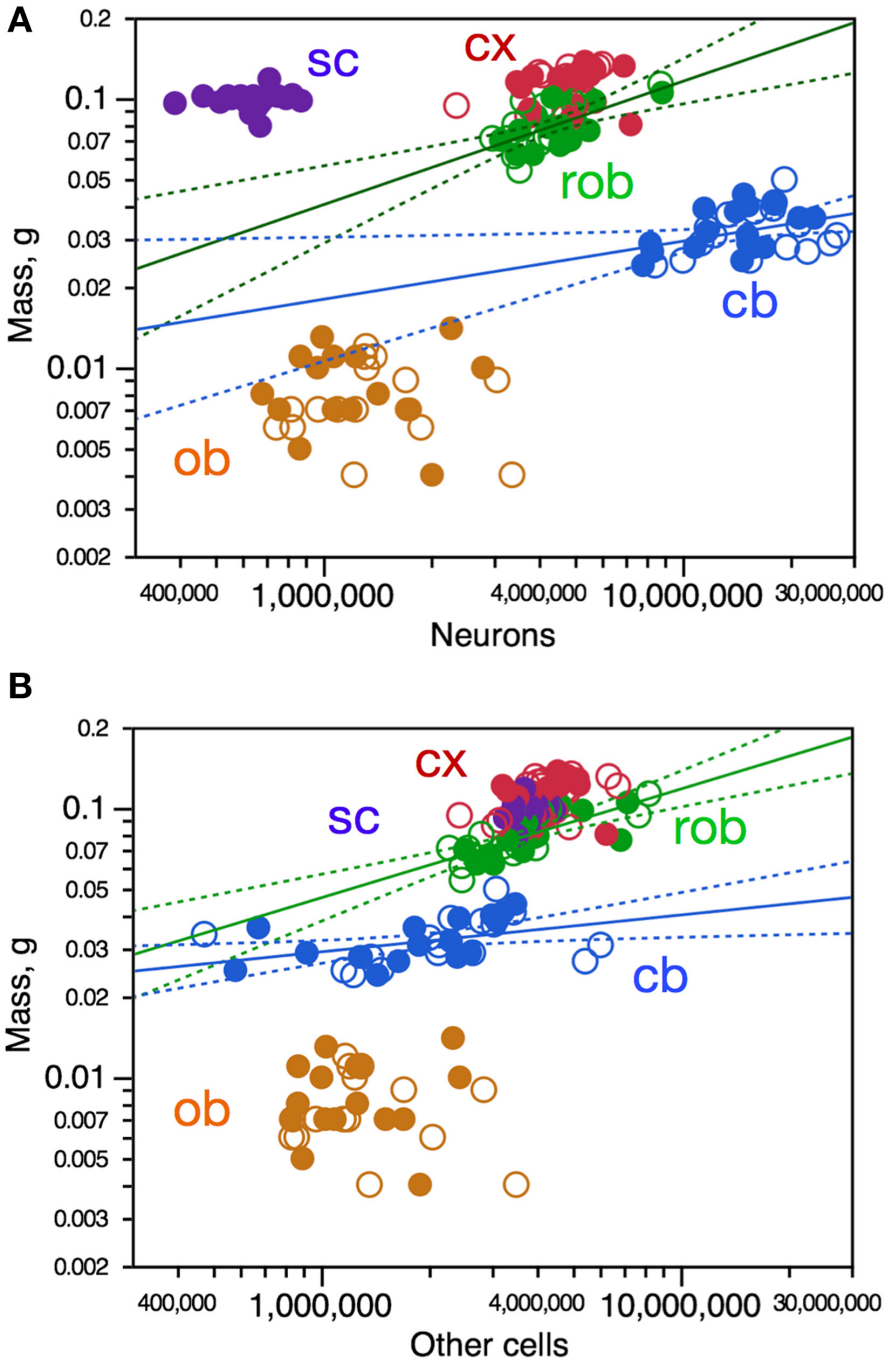
**Variations in CNS structure mass are poorly correlated, if at all, with variation in number of neurons **(A)** and number of other cells **(B)** across mouse individuals. Each data point represents one CNS structure in one individual**. Values for brain structures refer to right (filled symbols) and left sides (open symbols) separately. Cx, cerebral cortex (gray and white matter combined); cb, cerebellum; ob, olfactory bulb; rob, rest of brain; sc, spinal cord. **(A)** Spearman correlation coefficients for each structure: cx, ρ = 0.4062, *p* = 0.0210; rob, ρ = 0.5532, *p* = 0.0012; cb, ρ = 0.3375, *p* = 0.0547; ob, ρ = 0.0897, *p* = 0.6313; sc, ρ = 0.1286, *p* = 0.5999. Only the power functions with significant exponents are shown: cb, exponent, 0.216 ± 0.096, *r*^2^ = 0.140, *p* = 0.0322; rob, exponent, 0.458 ± 0.109, *r*^2^ = 0.380, *p* = 0.0002; cx, *p* = 0.2251; ob, *p* = 0.8675; sc, *p* = 0.7778. **(B)** Spearman correlation coefficients for each structure: cx, ρ = 0.3580, *p* = 0.0443; rob, ρ = 0.7499, *p* < 0.0001; cb, ρ = 0.5700, *p* = 0.0005; ob, ρ = 0.0690, *p* = 0.7122; sc, ρ = 0.3573, *p* = 0.1332. Only the power functions with significant exponents are shown: cb, exponent, 0.137 ± 0.054, *r*^2^ = 0.172, *p* = 0.0166; rob, exponent, 0.404 ± 0.072, *r*^2^ = 0.519, *p* < 0.0001; cx, *p* = 0.1228; ob, *p* = 0.6807; sc, *p* = 0.1332.

Variations in structure mass across individuals show some correlation with variations in numbers of neurons in the cerebral cortex (Spearman, ρ = 0.4062, *p* = 0.0210) and in the remaining areas (ρ = 0.5532, *p* = 0.0012), and did not reach significance in the cerebellum, olfactory bulb, or spinal cord (*p*-values in Figure 2A). Variations in structure mass however could only be described as significant power functions of numbers of neurons in the cerebellum and rest of brain, but with low *r*^2^-values of 0.140 and 0.380, respectively (Figure 2A, blue and green). Variations in the mass of some brain structures are thus somewhat correlated with variations in numbers of neurons across individuals, but poorly.

Variations in structure mass across individuals were also somewhat correlated with variations in numbers of other cells in the cerebral cortex (Spearman, ρ = 0.3580, *p* = 0.0443), in the cerebellum (ρ = 0.5700, *p* = 0.0005) and in the remaining areas (ρ = 0.7499, *p* < 0.0001), but not in the olfactory bulb nor in the spinal cord (Figure 2B). Again, variations in structure mass however could only be described as significant power functions of numbers of other cells in the cerebellum and remaining areas, again with low *r*^2^-values of 0.172 and 0.519, respectively (Figure 2B, blue and green). Variations in the mass of some brain structures are thus also correlated with variations in numbers of other cells across individuals, but poorly.

**Figure 3:**
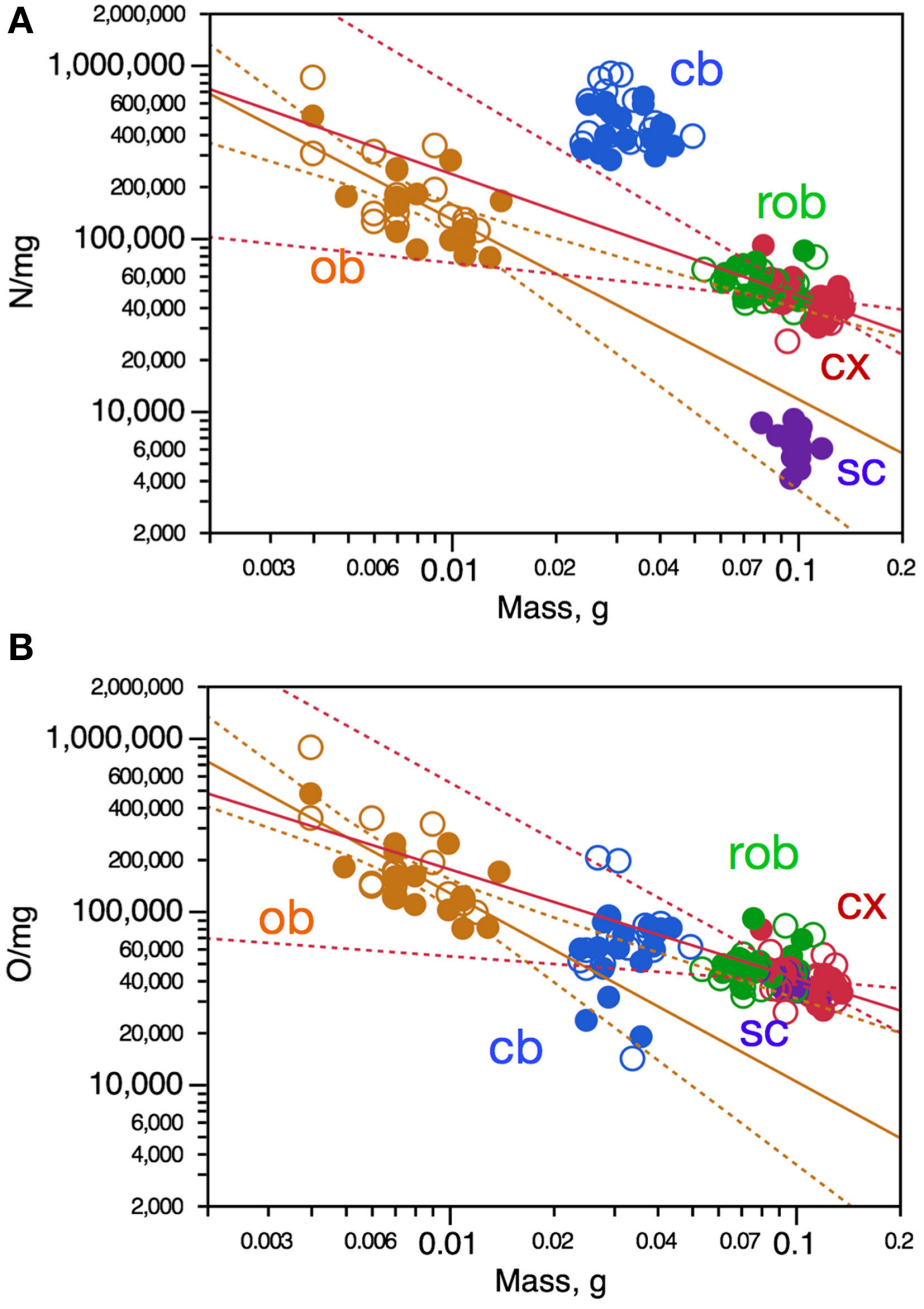
**Variation in neuronal density **(A)** and other cell density **(B)** is only poorly correlated with variation in CNS structure mass across mouse individuals**. Each data point represents one CNS structure in one individual. Values for brain structures refer to right (filled symbols) and left sides (open symbols) separately. Cx, cerebral cortex (gray and white matter combined); cb, cerebellum; ob, olfactory bulb; rob, rest of brain; sc, spinal cord. **(A)** Spearman correlation coefficients for each structure: ob, ρ = −0.5684, *p* = 0.0009; cx, *p* = 0.0802; rob, *p* = 0.1859; cb, *p* = 0.3777; sc, *p* = 0.3163. Only the power functions with significant exponents are shown: ob, exponent, −1.038 ± 0.229, *r*^2^ = 0.415, *p* < 0.0001; cx, exponent, −0.702 ± 0.240, *r*^2^ = 0.222, *p* = 0.0065; cb, *p* = 0.2308; rob, *p* = 0.3911; sc, *p* = 0.3163. **(B)** Spearman correlation coefficients for each structure: ob, ρ = −0.6446, *p* < 0.0001; cx, *p* = 0.0986; cb, *p* = 0.0950; rob, *p* = 0.6386; sc, *p* = 0.2126. Only the power functions with significant exponents are shown: ob, exponent, −1.087 ± 0.209, *r*^2^ = 0.482, *p* < 0.0001; cx, exponent, −0.626 ± 0.235, *r*^2^ = 0.191, *p* = 0.0123; cb, *p* = 0.6135; rob, *p* = 0.2248; sc, exponent, −0.705 ± 0.279, *p* = 0.0216.

To determine whether intraspecific variations in the mass of brain structures might be related to variations in the average size of the cells, we analyzed neuronal and other cell densities, which are proxies for the inverse of average neuronal and other cell size (including cell body and all axonal and dendritic arbors), respectively (Mota and Herculano-Houzel, 2014). Variations in neuronal density across individuals were significantly correlated with variations in structure mass only in the olfactory bulb, in which neuronal density varied across animals as a power function of olfactory bulb mass (Figure 3A, orange). Despite the lack of significant correlation, variations in neuronal density in the cerebral cortex across animals could be described as a power function of cortical mass, although with a small *r*^2^-of 0.222 (Figure 3A, red). Thus, larger olfactory bulbs and cerebral cortices tend to have smaller neuronal densities, and thus presumably larger neurons on average.

A similar pattern was found for other cell density, which also correlated with structure mass only in the olfactory bulb, in which other cell density varied as a power function of olfactory bulb mass with *r*^2^ = 0.482 (Figure 3B, orange). Other cell density also varied across animals as a power function of cortical mass, although again with a small *r*^2^ of only 0.191 (Figure 3B, red). Thus, larger olfactory bulbs and cortices tend to have both smaller neuronal and other cell densities, and thus presumably larger neurons and other cells on average. In other brain structures and in the spinal cord, however, structure mass is not correlated with changes in average density of neurons or other cells.

### Strong correlation between numbers of neurons and other cells

**Figure 4:**
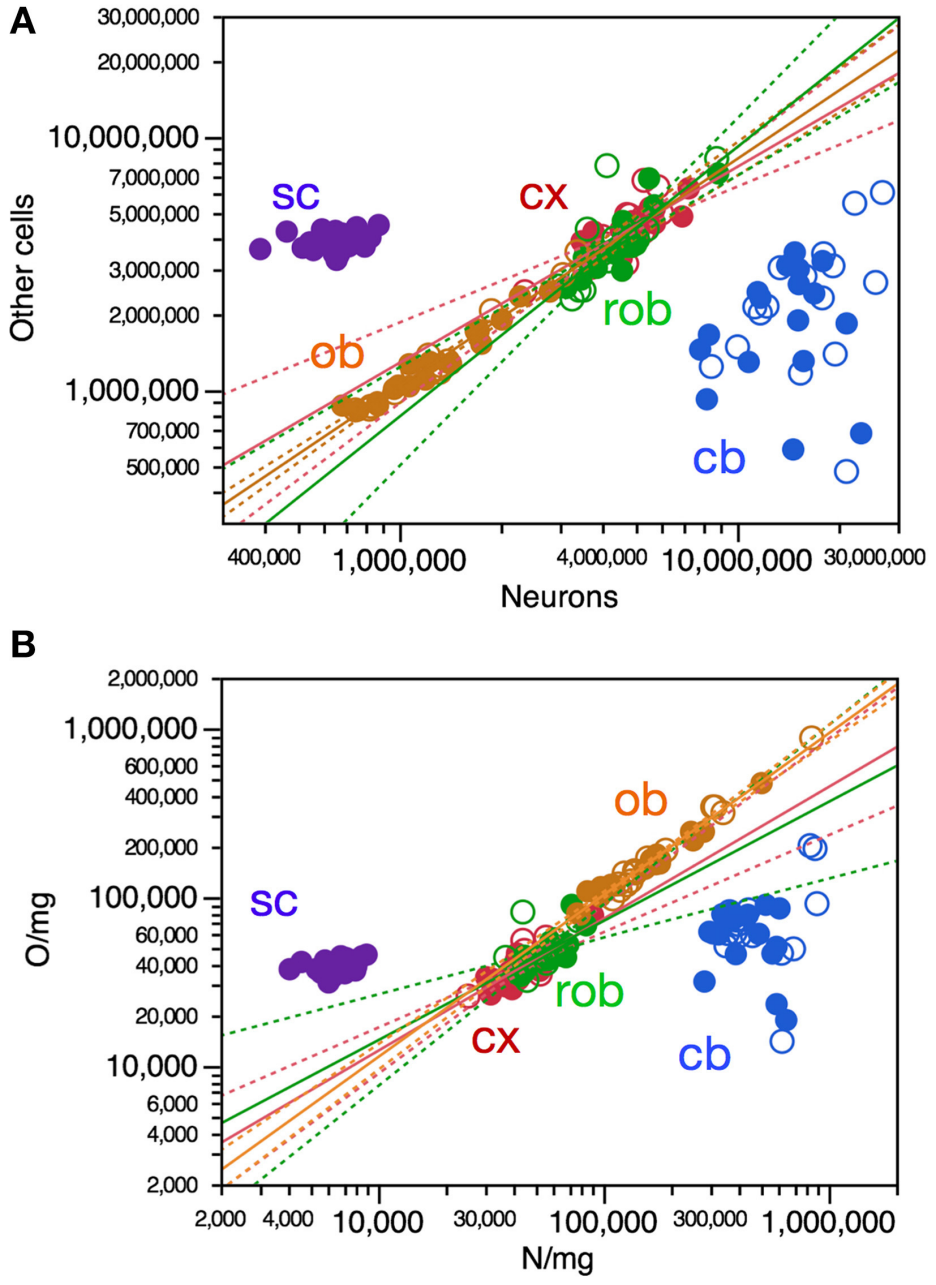
**Variations in numbers and density of other cells are strongly correlated with variation in numbers and density of neurons across mouse individuals**. Each data point represents one CNS structure in one individual. Values for brain structures refer to right (filled symbols) and left sides (open symbols) separately. Cx, cerebral cortex (gray and white matter combined); cb, cerebellum; ob, olfactory bulb; rob, rest of brain; sc, spinal cord. **(A)** Only the power functions with significant exponents are shown: cx, 0.774 ± 0.114, *r*^2^ = 0.606, *p* < 0.0001; ob, exponent, 0.897 ± 0.035, *r*^2^ = 0.956, *p* < 0.0001; rob, exponent, 1.065 ± 0.147, *r*^2^ = 0.645, *p* < 0.0001; cb, *p* = 0.1472; sc, *p* = 0.3503. **(B)** Only the power functions with significant exponents are shown: cx, exponent, 0.781 ± 0.102, *r*^2^ = 0.661, *p* < 0.0001; rob, exponent, 0.706 ± 0.176, *r*^2^ = 0.356, *p* = 0.0004; ob, exponent, 0.958 ± 0.030, *r*^2^ = 0.773, *p* < 0.0001; cb, *p* = 0.6824; sc, *p* = 0.1545.

In contrast to the weak correlations across structure mass and numbers of cells across individuals, we found a very strong correlation between numbers of neurons and numbers of other cells in three of the four brain structures (cerebral cortex, ρ = 0.7676, *p* < 0.0001; olfactory bulb, ρ = 0.9605, *p* < 0.0001; remaining areas, ρ = 0.7976, *p* < 0.0001; cerebellum, ρ = 0.3112, *p* = 0.0780; Figure 4A). As in the cerebellum, there was no significant relationship across individuals between numbers of neurons in other cells in the spinal cord (*p* = 0.3870; Figure 4A).

Variations in numbers of other cells across individuals can be described as power functions of numbers of neurons in the cerebral cortex, olfactory bulb and in the remaining areas. Remarkably, variations in numbers of other cells as a function of variations in numbers of neurons appear to be aligned across the cerebral cortex, olfactory bulb and remaining areas, yielding a joint Spearman correlation coefficient of 0.9322 (*p* < 0.0001) and a single power function joint exponent of 0.898 ± 0.023 (*p* < 0.0001, *r*^2^ = 0.942). Individual increases or decreases in numbers of neurons are thus strongly tied to increases or decreases in numbers of other cells in all brain structures except for the cerebellum, and also not in the spinal cord. Thus, individuals with more neurons in the cerebral cortex, olfactory bulb or rest of brain tend strongly to also have more other cells in these structures—although, as shown above, having more cells in each brain structure does not translate directly into larger brain structures.

### Increasing cell numbers compensated by increasing cell densities

**Figure 5:**
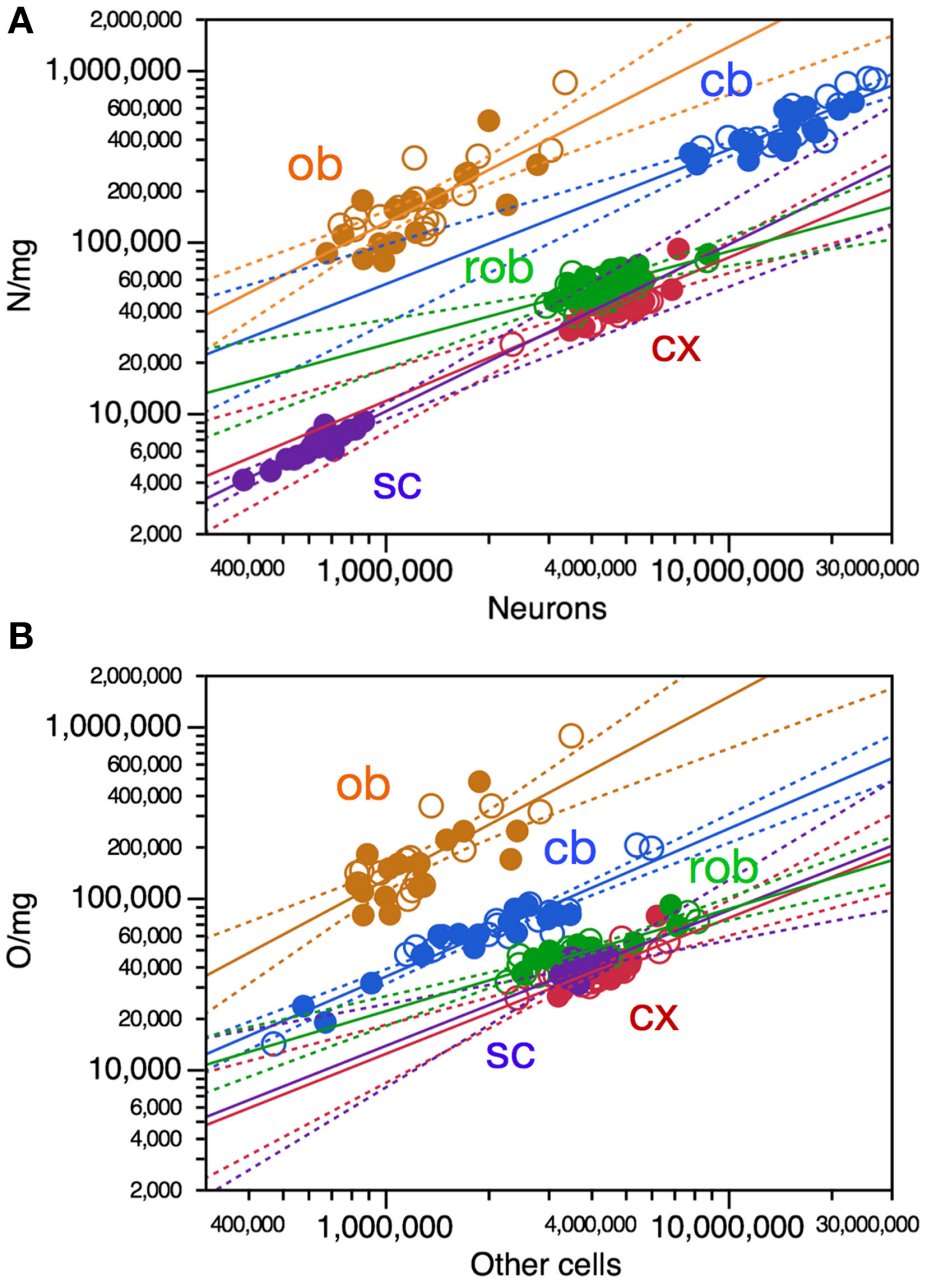
**Increasing numbers of neurons and other cells are strongly correlated with increasing cell density across mouse individuals**. Each data point represents one CNS structure in one individual. Values for brain structures refer to right (filled symbols) and left sides (open symbols) separately. Cx, cerebral cortex (gray and white matter combined); cb, cerebellum; ob, olfactory bulb; rob, rest of brain; sc, spinal cord. **(A)** All Spearman correlations have ρ > 0.5, *p* < 0.001. All power functions have significant exponents: cx, 0.836 ± 0.132, *r*^2^ = 0.572, *p* < 0.0001; cb, exponent, 0.784 ± 0.096, *r*^2^ = 0.682, *p* < 0.0001; ob, exponent, 1.025 ± 0.150, *r*^2^ = 0.616, *p* < 0.0001; rob, exponent, 0.542 ± 0.109, *r*^2^ = 0.461, *p* < 0.0001; sc, exponent, 0.972 ± 0.097, *r*^2^ = 0.856, *p* < 0.0001. **(B)** All Spearman correlations have ρ > 0.6, *p* < 0.001. All power functions have significant exponents: cx, 0.792 ± 0.131, *r*^2^ = 0.550, *p* < 0.0001; cb, exponent, 0.862 ± 0.054, *r*^2^ = 0.891, *p* < 0.0001; ob, exponent, 1.068 ± 0.164, *r*^2^ = 0.595, *p* < 0.0001; rob, exponent, 0.596 ± 0.072, *r*^2^ = 0.702, *p* < 0.0001; sc, exponent, 0.791 ± 0.198, *r*^2^ = 0.484, *p* = 0.0009.

One way in which variations in numbers of neurons and other cells across individuals might not result directly in variations in the mass of brain structures is if increasing cell numbers across individuals were almost entirely compensated by decreasing cell sizes, that is, increasing cell densities. We thus examined how variations in numbers of neurons and other cells correlate with variations in the density of these cells across structures. Indeed, in all CNS structures (including the cerebellum and spinal cord) we found that increasing numbers of neurons correlate with increased neuronal density (all correlations, ρ > 0.5, *p* < 0.001) that could also be described significantly as power functions (exponents in Figure 5A).

Increasing numbers of other cells were also strongly correlated with increased other cell density in all brain structures as well as in the spinal cord (all correlations, ρ >0.6, *p* < 0.0001) that could also be described significantly as power functions (Figure 5B). Notice that the cerebellum and remaining areas, the two structures with the smallest rates of increase in neuronal density as the numbers of these cells increase, are also the only structures whose mass was found to increase as a significant function of numbers of neurons across individuals (Figure 2A). The remaining areas, with the smallest rate of increase in other cell density as the number of these cells increases across individuals, is also one of two structures whose mass varies as a function of its number of other cells across individuals (Figure 2B). Thus, the increase in cell density strongly associated with larger numbers of cells (neuronal or not) across individuals almost entirely compensates for the increased mass that would otherwise ensue as a simple function of increased cells numbers if their average size did not change.

### Covariation of neuronal and other cell densities

In line with the concerted increase in numbers of other cells and neurons as well as with the concerted increase in numbers of cells and their respective densities in each brain structure across individuals, intraspecific variations in neuronal density were strongly correlated with variations in other cell density in the cerebral cortex (Spearman, ρ = 0.7896, *p* < 0.0001), olfactory bulb (Spearman, ρ = 0.9726, *p* < 0.0001) and remaining areas (Spearman, ρ = 0.7004, *p* < 0.0001; Figure 4B). In the cerebellum and spinal cord, where variations in number of neurons were not correlated with variations in numbers of other cells, we also found no significant correlation between neuronal density and other cell density (*p* = 0.8160 and *p* = 0.0928, respectively; Figure 4B). The significant correlations across individuals between neuronal density and other cell density within each brain structure could also be described as significant power functions in the cerebral cortex, remaining areas, and olfactory bulb (Figure 4B). This suggests that in these three brain structures, where variations in numbers of neurons are linked to variations in numbers of other cells, most of the intraspecific variation in average neuronal density, and thus average neuronal cell size, is associated with variation in average density of other cells, and thus the average size of non-neuronal cells. Remarkably, variations in other cell density as a function of neuronal density appear to be aligned across the cerebral cortex and remaining areas, yielding a joint correlation coefficient of 0.7715 (<0.0001) and a single power function joint exponent of 0.773 ± 0.082 (*p* < 0.0001, *r*^2^ = 0.590), as if a single relationship applied across these portions of the neuraxis (Figure 4B). In contrast, in the cerebellum and spinal cord, where variations across individuals in numbers of neurons are not correlated with numbers of other cells, average neuronal cell size is also not correlated with average other cell size. In all CNS structures, however, larger numbers of cells are correlated with larger densities and thus smaller cell size across individuals.

### Variation in O/N correlated with neuronal density

**Figure 6:**
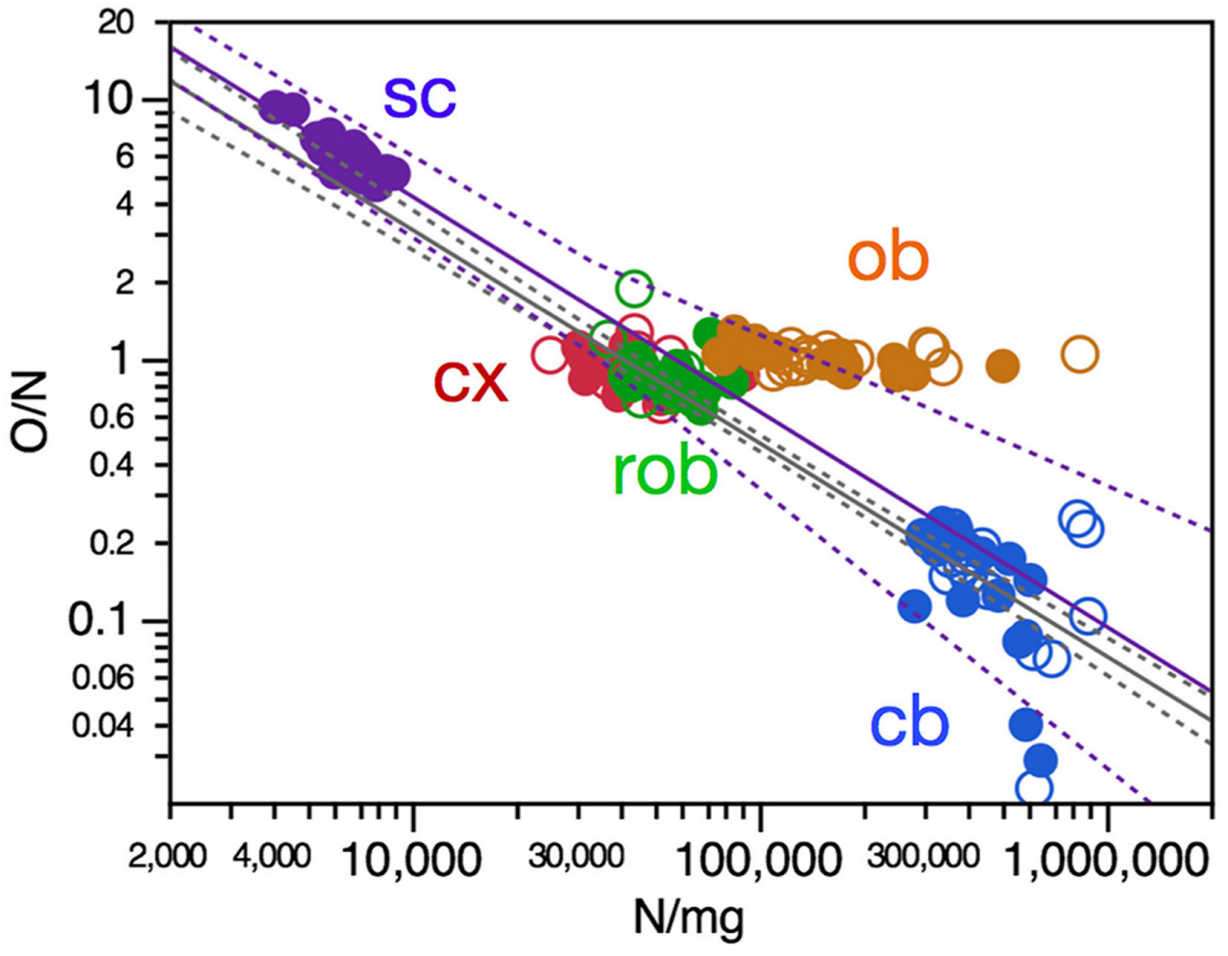
**O/N ratio in most CNS structures varies with neuronal density across mouse individuals**. Each data point represents one CNS structure in one individual. Values for brain structures refer to right (filled symbols) and left sides (open symbols) separately. Cx, cerebral cortex (gray and white matter combined); cb, cerebellum; ob, olfactory bulb; rob, rest of brain; sc, spinal cord. Power functions plotted are for the combined cerebral cortex, cerebellum and remaining areas (in gray; exponent, −0.818 ± 0.033, *r*^2^ = 0.866, *p* < 0.0001) and for the spinal cord (in purple; exponent, −0.826 ± 0.117, *r*^2^ = 0.745, *p* < 0.0001).

The ratio between numbers of other cells and numbers of neurons, O/N, has been found to vary both across brain structures (cerebral cortex, cerebellum, and remaining areas), across mammalian species (reviewed in Herculano-Houzel, 2014) as well as across regions of the human cerebral cortex (Ribeiro et al., 2013) as overlapping power functions of neuronal density, with negative exponents around −0.8. Here we find that, across mouse individuals, the ratio O/N also varies as a significant power function of neuronal density with an exponent of −0.818 ± 0.033 across the pooled cerebral cortex, cerebellum and remaining areas (Figure 6, gray), similar to the exponent found in the cerebellum (−0.833 ± 0.300, *p* = 0.0093, *r*^2^ = 0.199), and also in the spinal cord (Figure 6, purple), although the exponent does not reach significance within the remaining areas alone (*p* = 0.1059) and has a smaller value of −0.219 ± 0.102 within the cerebral cortex (*p* = 0.0402, *r*^2^ = 0.133).

### Correlations across structures

To determine whether individual variations in numbers of cells and cell density were also correlated across structures, we ran a pairwise Spearman correlation analysis across structures. Unexpectedly, we found that variations in neuronal density were positively correlated between the cerebral cortex and the cerebellum (ρ = 0.4768, *p* = 0.0077) and even more strongly between the cerebellum and olfactory bulb across individuals (ρ = 0.7024, *p* < 0.0001; the correlation between cerebral cortex and olfactory bulb did not reach significance, with *p* = 0.0892; Table 2). This suggests that neuronal densities increase (and therefore average neuronal size decreases) concertedly across the cerebellum and the olfactory bulb. Variations in neuronal density in the remaining areas were significantly, but negatively, correlated with variations in neuronal density in the spinal cord (ρ = −0.4378, *p* = 0.0138). No other correlations between neuronal densities were significant across structures (Table 2).

**Table 2:** **Correlations across structures**.

	**M_CX_**	**M_CB_**	**M_OB_**	**M_RA_**	**M_SC_**	**N_CX_**	**N_CB_**	**N_OB_**	**N_RA_**	**N_SC_**	**DN_CX_**	**DN_CB_**	**DN_OB_**	**DN_RA_**	**DN_SC_**
M_CX_															
M_CB_	0.4119 *P* = 0.0191														
M_OB_	n.s. *P* = 0.0620	n.s. *P* = 0.3464													
M_RA_	−0.3937 *P* = 0.0234	−0.4063 *P* = 0.0210	n.s. *P* = 0.3680												
M_SC_	n.s. *P* = 0.8006	0.3595 *P* = 0.0399	n.s. *P* = 0.8772	n.s. *P* = 0.2704											
N_CX_	0.4062 *P* = 0.0210	n.s. *P* = 0.0776	n.s. *P* = 0.4916	n.s. *P* = 0.5779	n.s. *P* = 0.1495										
N_CB_	−0.3600 *P* = 0.0430	n.s. *P* = 0.0547	n.s. *P* = 0.3439	n.s. *P* = 0.7106	n.s. *P* = 0.1979	n.s. *P* = 0.1782									
N_OB_	−0.5052 *P* = 0.0044	n.s. *P* = 0.1920	n.s. *P* = 0.6313	n.s. *P* = 0.5724	n.s. *P* = 0.6858	n.s. *P* = 0.8000	0.4734 *P* = 0.0071								
N_RA_	n.s. *P* = 0.2240	−0.4603 *P* = 0.0105	n.s. *P* = 0.5000	0.5532 *P* = 0.0012	n.s. *P* = 0.8944	n.s. *P* = 0.3801	n.s. *P* = 0.3510	n.s. *P* = 0.4479							
N_SC_	n.s. *P* = 0.5029	n.s. *P* = 0.0663	n.s. *P* = 0.3586	n.s. *P* = 0.4387	n.s. 0.4418	0.3796 *P* = 0.0321	n.s. *P* = 0.0559	n.s. *P* = 0.7552	−0.4820 *P* = 0.0060						
DN_CX_	n.s. *P* = 0.0802	n.s. *P* = 0.9190	n.s. *P* = 0.5007	n.s. *P* = 0.3336	n.s. *P* = 0.3336	0.6664 *P* < 0.0001	0.4656 *P* = 0.0095	n.s. *P* = 0.0553	n.s. *P* = 0.8649	n.s. *P* = 0.2586					
DN_CB_	−0.6082 *P* = 0.0002	n.s. *P* = 0.3777	n.s. *P* = 0.1521	n.s. *P* = 0.2830	n.s. *P* = 0.2830	n.s. *P* = 0.7802	0.8269 *P* < 0.0001	0.6274 *P* = 0.0002	n.s. *P* = 0.8181	n.s. *P* = 0.3088	0.4768 *P* = 0.0077				
DN_OB_	−0.7012 *P* < 0.0001	n.s. *P* = 0.0672	−0.5684 *P* = 0.0009	n.s. *P* = 0.4778	n.s. *P* = 0.4778	n.s. *P* = 0.4709	0.4859 *P* = 0.0056	0.7218 *P* < 0.0001	n.s. *P* = 0.8272	n.s. *P* = 0.7282	n.s. *P* = 0.0892	0.7024 *P* < 0.0001			
DN_RA_	n.s. *P* = 0.2451	n.s. *P* = 0.2663	0.4340 *P* = 0.0166	n.s. *P* = 0.1859	n.s. *P* = 0.1859	n.s. *P* = 0.6243	n.s. *P* = 0.0846	n.s. *P* = 0.5592	0.5810 *P* = 0.0006	−0.4866 *P* = 0.0055	n.s. *P* = 0.1158	n.s. *P* = 0.0832	n.s. *P* = 0.5310		
DN_SC_	n.s. *P* = 0.5780	n.s. *P* = 0.1563	n.s. *P* = 0.3331	n.a. *P* = 0.2545	n.s. *P* = 0.1417	n.s. *P* = 0.1702	n.s. 0.1609	n.s. 0.7112	−0.4796 0.0063	0.8702 *P* < 0.0001	n.s. *P* = 0.5708	n.s. *P* = 0.2601	n.s. *P* = 0.6721	−0.4378 *P* = 0.0138	

Spearman correlation coefficient r is indicated where *P* < 0.05. Significant correlations within a same structure are indicated in dark gray. Significant correlations across structures are indicated in light gray. For clarity, correlations are shown only once for each pair, but all significant correlations are indicated by shading.

As shown above, all structures had strongly and positively correlated neuronal density and number of neurons across individuals (Figure 5A; Table 2). Additionally, increasing numbers of neurons in the cerebellum were significantly- and positively correlated with neuronal densities in the cerebral cortex and in the olfactory bulb, and conversely, neuronal density in the cerebellum was significantly and positively correlated with numbers of neurons in the olfactory bulb (Table 2). The correlations between cerebellum and olfactory bulb can be associated with the concerted variation in numbers of neurons in the cerebellum and olfactory bulb reported above. On the other hand, increasing numbers of neurons in the spinal cord were negatively correlated with neuronal density in the rest of brain, and vice-versa (Table 2).

Larger cerebral cortices were found to correlate strongly with smaller neuronal densities in the cerebellum and in the olfactory bulb across individuals (ρ = −0.6082 and −0.7012, respectively; *p* = 0.0002 and *p* < 0.0001), and also with fewer neurons in these structures (Table 2). Additionally, larger olfactory bulbs correlated with larger neuronal densities in the remaining areas (ρ = 0.4340, *p* = 0.0166). No other correlations between the mass of one structure and neuronal density in another structure were found to be significant (Table 2).

Individuals with larger cerebral cortical mass showed a weak tendency to also have a larger cerebellum (ρ = 0.4119, *p* = 0.0191) and smaller remaining areas (ρ = −0.3937, *p* = 0.0234; Table 2). Importantly, and in contrast, these correlations of structure mass across these brain structures were not accompanied by similar correlations in numbers of neurons across structures, in agreement with our finding that variations in structure mass are only poorly correlated with variations in their respective number of neurons across individuals. We only found significant positive correlations between numbers of neurons in the cerebellum and in the olfactory bulb across individuals (ρ = 0.4734, *p* = 0.0071) and between the cerebral cortex and spinal cord (ρ = 0.3796, *p* = 0.0321), and a negative correlation between numbers of neurons in the rest of brain and in the spinal cord (ρ = −0.4820, *p* = 0.0060; Table 2). That is, individuals with more neurons in the cerebral cortex showed no significant tendency to also have more neurons in the cerebellum, or fewer in the remaining areas, although they tended to have fewer neurons in the spinal cord. No other significant correlations were found between numbers of neurons in different structures across individuals (Table 2).

## Discussion

Here we show for the first time that variations in CNS structure mass do not directly reflect variations in numbers of neurons or other cells across adult individuals of a similar age. That is, mouse individuals with larger brains or CNS structures do not necessarily have more neurons than individuals with smaller brains or structures. Rather, our most remarkable finding is that, in all CNS structures, increased numbers of neurons across individuals are accompanied by increased neuronal densities, and therefore by smaller average neuronal cell size (including the soma and all dendritic and axonal arbors; Mota and Herculano-Houzel, 2014). Similarly, increased numbers of other cells across individuals in each CNS structure are accompanied by increased other cell densities, that is, smaller average other cell size in the structure. Thus, whatever mass might be gained by the addition of neurons and other cells to CNS structures seems to be largely compensated by a decrease in the average size of the cells across individuals. This trade-off between numbers of cells and average cell size explains how variations across individuals in number of cells (or cell densities) are only loosely correlated with variations in brain mass, if at all. Additionally, in the cerebral cortex, olfactory bulb and rest of brain, increased numbers of neurons are also accompanied by increased numbers of other cells, and neuronal and other cell densities vary coordinately across individuals in these structures, which implies the existence of common mechanisms controlling numbers of cells and average cell size, as discussed below.

Out of necessity, the present analysis was necessarily limited to an arbitrary division of brain structures into cerebral cortex/cerebellum/olfactory bulb/rest of brain, and of all cells into simply neurons and other cells. While these simple categories certainly do not reflect the enormous complexity of brain structures and the various cell subtypes, we find it striking that very strong correlations still emerge for neurons as a whole, other cells (glial and vascular) as a whole, and for something as diverse as the “rest of brain” that certainly includes many functional and structurally different regions. These strong correlations across individuals between numbers of cells and cell densities, and between numbers of neurons and glial cells, indicate that very basic mechanisms must be at play in the joint determination of numbers of cells and average cell size, possibly well before the characteristic complexity of brain tissue emerges in development.

Although we only examined adult, male animals, our data strongly indicate the existence of two fundamental (but not necessarily genetic) regulatory mechanisms in CNS development: One that ties final numbers of other cells to numbers of neurons (which are achieved first in development; Bandeira et al., 2009) in all structures except the cerebellum and spinal cord; and another that ties average cell size to numbers of cells generated in all CNS structures, such that the more cells generated (either neurons or other cells), the smaller they are on average. Our data also indicate that the same mechanism associates numbers of cells to cell size across neurons and other cells alike, given that variations in neuronal density were found to be tightly linked to variations in other cell density across individuals in all structures whenever numbers of neurons were also tied to numbers of other cells in the structure.

One possible scenario to explain how more cells are tied to smaller average cell size is that final structure mass is somehow a limiting factor to cell size, so that any larger numbers of neurons also have to be smaller to fit a same volume (or mass, measured here). This, however, seems unlikely for three main reasons. First, brain mass is a dependent variable that is necessarily the result of the product of numbers of cells and their average mass achieved during the process of development. As such, brain mass cannot be a controlling parameter that influences, much less determines, other variables. It could still be argued that brain mass is constrained by skull volume, which was not measured here, but the existence of conditions such as hydrocephaly, where the skull becomes deformed, indicates that skull volume does not constrain brain volume. Second, we find that brain (and spinal cord) structure mass is not invariant, but rather varies across individuals by 1.3- to 1.9-fold (depending on the structure). This variation argues against an internal or external constraint to brain mass. Finally, principal component analysis shows that structure mass does not load in the first component, but rather only in the second, after numbers of neurons and neuronal density. Thus, structure mass is unlikely to be a limiting or determinant factor to numbers of cells and average cell size in any way. Rather, structure mass appears to be the consequence of the combination of numbers of cells and their average size, which are somehow tied together.

In contrast to the strong correlation between numbers of cells and average cell mass within each structure, we found only weak and occasional evidence of concerted variations in the allocation of numbers of neurons across structures, and of average cell size across structures, suggesting that numbers of neurons are regulated and allocated independently to different brain structures. Importantly, we found no evidence of compensatory changes in the distribution of neurons across brain structures, which would happen if a limited pool of neurons were allocated to the different brain structures: not only is there no negative correlation between numbers of neurons in the different brain structures (except between the rest of brain and the spinal cord), but variation in numbers of neurons across individuals actually exceeds variation in brain mass in each CNS structure. Evolutionarily, the lack of concerted changes in cell numbers between brain structures across individuals offers a substrate for mosaic evolution (Barton and Harvey, 2000), as structures appear quite free from one another to vary in numbers of neurons across individuals.

Intriguingly, the lack of concerted variation in numbers of neurons between brain structures across individuals appears at first glance to be at odds with the concerted scaling of numbers of neurons across the cerebral cortex, cerebellum and rest of brain across species (Herculano-Houzel et al., 2014). Similarly, the finding that more neurons are linked to *larger* neuronal densities across individuals is also the opposite of what is found in evolution, where larger numbers of neurons in brain structures are accompanied, in variable degrees, by smaller neuronal densities across species (Haug, 1987; Herculano-Houzel et al., 2014). It is interesting to notice that a similar discrepancy has been found in how the volume of the cerebral cortex scales across species with increasing volume of the medulla (hypermetrically, that is, with an allometric exponent above 1.3) and how it scales across human individuals (hypometrically, that is, with an allometric exponent smaller than 0.8)—although the authors of that study mistakenly interpreted the presence of an allometric relationship across individuals as the source of an allometric relationship across species in the opposite direction (Charvet et al., 2013). Likewise, the variation found here across individuals cannot serve as a direct source of evolutionary variation across species—or species with larger brain structures, with more neurons, would also have smaller, and not larger, neurons (Herculano-Houzel et al., 2014).

**Figure 7:**
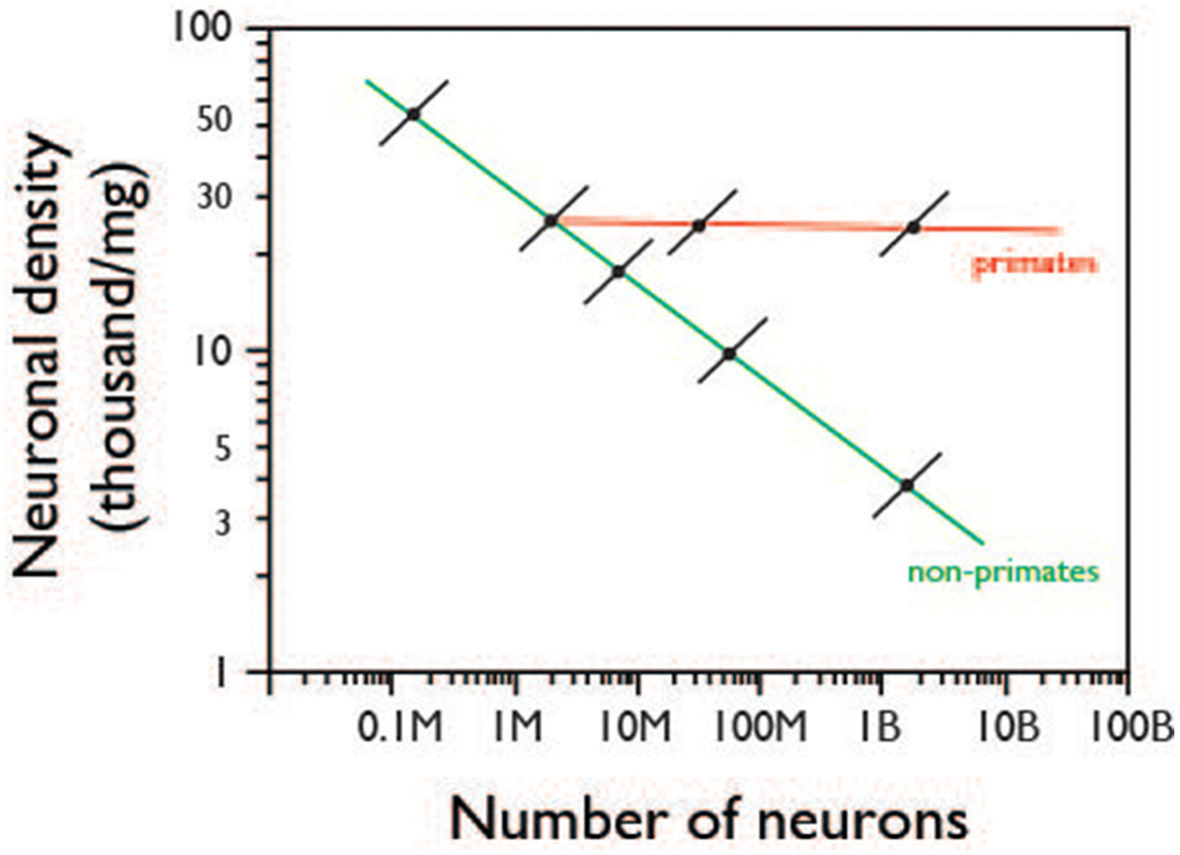
**Proposed relationship between intraspecific and interspecific scaling of neuronal density with number of neurons**. Colored lines indicate allometric relationships across species, as reviewed in Herculano-Houzel et al. (2014); Black lines indicate proposed allometric relationship within species, based on the present data. The diagram shows that across individuals of a same species (black lines), increasing numbers of neurons in CNS structures are accompanied by increasing neuronal densities (and thus smaller average cell size). In contrast, increasing numbers of neurons across species are accompanied by decreased neuronal density (and thus larger average neuronal cell size) in non-primate mammals (green line) or by relatively unchanging neuronal density (and average neuronal cell size) in primates (red line).

In other words, the data provided here for mouse individuals strongly indicates that cellular brain allometry across species is not simply an extension of cellular brain allometry across individuals of a same species—as was already known for brain mass vs. body mass allometry (Armstrong, 1990). Thus, the evolution of larger brains must not occur through the gradual positive selection of individuals with more and *smaller* neurons, but rather through the positive selection for step changes that lead to more and *larger* neurons, as illustrated in Figure 7.

Given the concerted addition of numbers of neurons to the cerebral cortex, cerebellum and rest of brain in mammalian evolution (Herculano-Houzel et al., 2014), despite the lack of strong correlations across these structures at the individual level shown here, we can postulate that whatever evolutionary mechanisms have led to step increases in numbers of neurons in the cerebral cortex has also caused concerted step increases (not necessarily by the same factor) in numbers of neurons in the rest of brain and cerebellum (Herculano-Houzel et al., 2014). These are likely to be genetic factors that impact multiple structures simultaneously, either directly or through the generation of larger initial numbers of neurons that are then matched across structures, possibly by activity-dependent, self-organizing mechanisms. Similarly, we can postulate that these mechanisms leading to step increases in numbers of neurons across species also cause these steps to be tied to an increased (and not decreased) average cell size, as illustrated in Figure 7. Such a quantitative link between increased number of neurons and increased average neuronal cell size was identified recently (Herculano-Houzel et al., 2014). Again, these mechanisms tying step increases in numbers of neurons to larger average cell size may or may not be genetic, as detailed below. Our data thus open new venues for inquiries into the genetic mechanisms that control cell size in conjunction with numbers of cells, and cell numbers across CNS structures.

Interestingly, one need not invoke a genetic mechanism to tie numbers of glial cells to numbers of neurons, as these numbers might be self-organized depending on the total neuronal mass in the tissue—provided that there is a mechanism that ties small variations in average glial cell size to larger variations in neuronal cell size across species (Mota and Herculano-Houzel, 2014). Similarly, we speculate that larger numbers of cells might lead to smaller cell size also through a self-organized, non-genetic mechanism whereby the faster pace of division of progenitor cells that leads to increased numbers of neurons or glial cells across individuals also causes the size of daughter cells to be smaller, which might limit the final size of the adult cells if it is associated with decreased levels of size-limiting molecules. However, in this same scenario, step increases in the size of progenitor cells (likely due to genetic changes) would lead to the roughly constant or even increased average neuronal cell size that is found to accompany increased numbers of neurons across primate and non-primate species, respectively, but not within a species.

Our finding that mice with larger brains (or larger CNS structures) do not have significantly more neurons than mice with smaller brains in the population argues that whatever mechanism leads to the evolution of species with larger brains with more neurons acts at the individual and population levels through selection for either larger brain size or larger numbers of neurons across individuals, but not both (as they are not linked across individuals of a same species). It is only once that evolutionary mechanisms such as those postulated above have led to a step increase in numbers of neurons, tied to an increase in average neuronal cell size, that larger numbers of neurons become correlated with larger brain size at the cross-species level. The finding of concerted scaling of numbers of neurons and average neuronal cell size across several orders of magnitude of brain mass in non-primate brains (Herculano-Houzel et al., 2014) indicates that whatever mechanism ties step increases in numbers of neurons to step increases in average neuronal cell size across species must have been conserved in evolution over at least the last 90 million years.

Finally, our finding that variations in brain structure mass are only poorly associated with variations in number of neurons across individuals, if at all, opens the possibility that the weak correlations found between brain size and cognitive abilities in humans (Luders et al., 2009) are due to a main correlation with numbers of neurons instead, which are not translated directly into brain size. We are currently examining that possibility.

### Conflict of interest statement

The authors declare that the research was conducted in the absence of any commercial or financial relationships that could be construed as a potential conflict of interest.
